# *TIME FOR COFFEE* controls root meristem size by changes in auxin accumulation in *Arabidopsis*

**DOI:** 10.1093/jxb/ert374

**Published:** 2013-11-25

**Authors:** Li-Wei Hong, Da-Wei Yan, Wen-Cheng Liu, Hong-Guo Chen, Ying-Tang Lu

**Affiliations:** ^1^College of Life Sciences, Wuhan University, Wuhan 430072, PR China; ^2^College of Chemistry and Biology, Hubei University of Science and Technology, Xianning 437100, Hubei Province, PR China

**Keywords:** *Arabidopsis thaliana*, auxin, circadian clock, jasmonic acid, *MYC2*, *PIN*, root meristem, *TIME FOR COFFEE*.

## Abstract

Roots play important roles in plant survival and productivity as they not only anchor the plants in the soil but are also the primary organ for the uptake of nutrients from the outside. The growth and development of roots depend on the specification and maintenance of the root meristem. Here, we report a previously unknown role of *TIME FOR COFFEE* (*TIC*) in controlling root meristem size in *Arabidopsis*. The results showed that loss of function of *TIC* reduced root meristem length and cell number by decreasing the competence of meristematic cells to divide. This was due to the repressed expression of *PIN* genes for decreased acropetal auxin transport in *tic-2*, leading to low auxin accumulation in the roots responsible for reduced root meristem, which was verified by exogenous application of indole-3-acetic acid. Downregulated expression of *PLETHORA1* (*PLT1*) and *PLT2*, key transcription factors in mediating the patterning of the root stem cell niche, was also assayed in *tic-2.* Similar results were obtained with *tic-2* and wild-type plants at either dawn or dusk. We also suggested that the *MYC2-*mediated jasmonic acid signalling pathway may not be involved in the regulation of *TIC* in controlling the root meristem. Taken together, these results suggest that *TIC* functions in an auxin–*PLT*s loop for maintenance of post-embryonic root meristem.

## Abstract (precis)

Reduced root meristem length in *tic* mutant is due to the repressed expression of *PIN* genes for decreased acropetal auxin transport, leading to low auxin accumulation. *MYC2*-mediated JA-signaling pathway may not be involved in this process

## Introduction

Plant growth is the result of the division and expansion of cells derived from localized meristem, which is composed of stem cell-like cells that are the precursors of all differentiated cell types (Laux and Mayer, 1998). Meristematic activity can be altered by versatile developmental and environmental cues during post-embryonic growth.

Individual cells have to coordinate their behaviours by means of small signalling molecules to form correctly patterned tissues in multicellular organisms. Auxin directional transport, as a unique mechanism, regulates the cell polarity and tissue development, and thus acts upon many aspects of plant growth and development (Feraru and Friml, 2008). It has been documented that root development requires shoot-derived transport of auxin and is dependent on the establishment of a gradient of auxin concentration (Friml *et al.*, 2003; Wisniewska *et al.*, 2006).

Auxin gradients can prolong or shorten the distinct phases of proliferation and differentiation, and auxin carriers are essential for these processes (Blilou *et al.*, 2005; Grieneisen *et al.*, 2007). PIN-FORMED (PIN) proteins have been proposed to be central rate-limiting components among auxin carriers, exhibiting asymmetric plasma membrane localization and determining the polarity of auxin transport (Wisniewska *et al.*, 2006; Sassi *et al.*, 2012). The polarity and amount of PIN proteins decide the polar auxin transport direction and amplitude, and the activity of PINs can be modulated by endogenous signals to trigger developmental decisions (Krecek *et al.*, 2009).

In *Arabidopsis* roots, cell-to-cell auxin transport is modulated by at least five *PIN*s, namely *PIN1*, *PIN2*, *PIN3*, *PIN4*, and *PIN7*, in different or overlapping groups of cells, maintaining an auxin maximum and concentration gradient in the root apex (Blilou *et al.*, 2005; Dello Ioio *et al.*, 2008; Lin *et al.*, 2012). Downregulation of the expression of *PIN*s in the root meristem or mutants lacking multiple *PIN* genes result in promoted cell differentiation and a reduced meristem size (Vieten *et al.*, 2005).

It has been documented that both *SHORT-ROOT* (*SHR*)/*SCARECROW* (*SCR*) and *PLETHORA*-mediated parallel pathways are required for the maintenance of the root stem cell niche. *SHR* and *SCR*, two members of the GRAS family of transcription factors, provide positional information to specify the identity of the quiescent centre (QC) and regulate the functions of the associated stem cells in the root (Helariutta *et al.*, 2000; Lim *et al.*, 2000). The *PLETHORA* genes *PLT1* and *PLT2* are one conduit for auxin action. Their abundance is dependent on auxin-regulated auxin response factors (Aida *et al.*, 2004; Dhonukshe *et al.*, 2012), but crucially, the expression of *PIN* genes is contingent on *PLETHORA* activity (Galinha *et al.*, 2007; Prasad *et al.*, 2011), revealing a feedback control between auxin and the *PLETHORA* factors. Furthermore, *PLT* gene dosages define different outputs establishing the position of the QC, from stem cell identity to mitotic activity and cellular differentiation (Galinha *et al.*, 2007).

Another phytohormone, jasmonic acid (JA), is also reported to inhibit primary root growth (Chen *et al.*, 2011*b*). This hormone, perceived by the CORONATINE INSENSITIVE 1 (COI1) receptor, promotes degradation of JASMONATE-ZIM-DOMAIN (JAZ) proteins and frees the transcriptional regulation activity of MYC2, the major transcription factor of JA-mediated gene expression (Kazan and Manners, 2013). It has been shown that JA regulates root stem cell niche maintenance and meristem activity in a *MYC2*-dependent manner, in which MYC2 binds directly to the promoters of *PLETHORA* genes to suppress their expression in response to JA (Chen *et al.*, 2011*b*).

A recent report demonstrated that MYC2 interacts with TIME FOR COFFEE (TIC), which acts as a negative regulator in JA signalling by repressing MYC2 protein accumulation (Shin *et al.*, 2012). *TIC* functions as a circadian clock regulator to maintain circadian period and amplitude, although its transcriptional and translational levels are constantly present over diurnal time (Hall *et al.*, 2003; Ding *et al.*, 2007). Plants have adapted their physiology, environmental responsiveness, and development to make use of the diurnal light/dark cycle (McClung, 2006). For example, root elongation rates exhibit an oscillation (Fisahn *et al.*, 2012). Also, the transcription of many genes shows clock regulation in the DIURNAL database (Mockler *et al.*, 2007). Thus, it would be interesting to test whether *TIC* is also involved in root development.

Our data uncovered a previously unknown role of *TIC* in controlling root meristem size by regulating auxin accumulation in *Arabidopsis*. Our study showed that *TIC* was highly expressed in root meristem, and that disruption of *TIC* led to a decrease in the size and cell number of root meristem. Mutation of *TIC* repressed the expression of *PIN1*, *PIN2*, *PIN3*, and *PIN7* for low auxin accumulation, resulting in downregulation of *PLT1/2* expression at both dawn and dusk. Furthermore, our experiments also suggested that *MYC2* may not be involved in *TIC*-mediated regulation of root meristem.

## Materials and methods

### Plant material and growth conditions

*Arabidopsis thaliana* ecotype Columbia (Col-0) was used as the wild type. The plant materials used in this study have been described previously: *CycB1;1::GUS* (Colon-Carmona *et al.*, 1999); *QC25::GUS* (Chen *et al.*, 2011*b*); *DR5::GFP* (Benkova *et al.*, 2003); *PIN1::PIN1-GFP* (Benkova *et al.*, 2003); *PIN2::PIN2-GFP* (Blilou *et al.*, 2005); *PIN3::PIN3-GFP* (Blilou *et al.*, 2005); *PIN7::PIN7-GFP* (Blilou *et al.*, 2005); and *PLT1::ERCFP* (Xu *et al.*, 2006). Seeds of *tic-2* (SAIL_753_E03) (Hall *et al.*, 2003) and *myc2-1* (SALK_040500) (Boter *et al.*, 2004) were obtained from the Arabidopsis Biological Resource Center.

Seeds were surface sterilized for 5min in 5% commercial kitchen bleach, washed three times with sterile water, and plated on half-strength Murashige and Skoog (MS) medium (pH 5.8) (Sigma-Aldrich) with 1% sucrose and 1% agar. Plants were stratified at 4 °C for 3 d in the dark and then transferred to a phytotron, set at 23 °C under a light intensity of 80mM photons m^–2^ s^–1^ in vertically oriented Petri dishes. The photoperiod was a 12h light/12h dark (12L/12D) cycle.

### Vector constructs and transgenic lines

A 2691bp genomic fragment upstream of the translation start codon was PCR amplified and inserted into the *Sma*I sites of the binary vector pBI101.2 (Jefferson *et al.*, 1987), resulting in transcriptional fusion of the *TIC* promoter with the β-glucuronidase (GUS) coding region. This construct was sequenced and transformed into *A. thaliana* (Col-0) by *Agrobacterium tumefaciens* strain pGV3101 using the floral dip method (Clough and Bent, 1998). The specific primers for the *TIC* promoter were TIC-proF and TIC-proR, listed in Supplementary Table S1 at *JXB* online.

### GUS staining

GUS staining was carried out as described previously (Hu *et al.*, 2010). Briefly, seedlings were incubated at 37 °C in staining solution [100mM sodium phosphate buffer, pH 7.5, containing 10.0mM EDTA, pH 8.0, 0.5mM K_3_(Fe[CN]_6_) and 0.5mM K_4_(Fe[CN]_6_), 0.1% Triton X-100 and 1.0mM 5-bromo-chloro-3-indolyl-β-d-glucuronide]. The GUS staining time was dependent on the transgenic marker lines: 1h for *DR5::GUS*, 6h for *QC25::GUS*, 12h for *CycB1;1::GUS*, and 15min for *TIC::GUS*.

### Root meristem size measurement

Seeds were germinated on half-strength MS medium containing 1% sucrose and 1% agar and grown in a vertical position. The number of root meristem cells was defined by counting the number of cells in a file extending from the initial cell adjacent to the QC to the first elongated cell in the cortex layer (Dello Ioio *et al.*, 2007). Results presented are averages of more than 30 seedlings and experiments were repeated at least three times.

### Microscopic analysis

For phenotypic analysis of root or GUS staining microexamination, seedlings were cleared and mounted with clearing solution (8g of chloral hydrate, 2ml of water, and 1ml of glycerol) on glass slide. The slides were examined under a differential interference contrast (DIC) Olympus BX60 microscope and photographed using a charge coupled device (CCD) Olympus DP72.

Confocal microscopy was performed using an Olympus FluoView 1000 confocal laser-scanning microscope according to the manufacturer’s instructions. Green fluorescent protein (GFP) and cyan fluorescent protein (CFP) lines were mounted with 20 µg ml^–1^ of propidium iodide (PI).

### RNA extraction and expression analysis

RNA extraction was performed using PureLink™ Plant RNA Reagent (Invitrogen) according to the instruction manual. All RNA samples were treated with RQ1 RNase-free DNase I (Promega) to remove DNA contamination and reverse transcription was carried out using ReverTra Ace^®^ (Toyobo).

Quantitative real-time (RT)-PCR assay was performed using a CFX96™ Real-Time PCR Detection System (Bio-Rad). *UBQ1* (AT3G52590) and PP2A subunit *PDF2* (AT1G13320) were chosen as reference genes using geNorm software (Vandesompele *et al.*, 2002; Czechowski *et al.*, 2005). PCR was performed as follows: 3min at 95 °C, followed by 40 cycles of denaturation for 15 s at 95 °C, annealing for 15 s at 58 °C, and extension for 20 s at 72 °C. All experiments were performed with three independent biological replicates and three technical repetitions. The specific primers used are listed in Supplementary Table S1.

### [^3^H]indole-3-acetic acid (IAA) transport assays

Auxin transport assays were conducted according to a method described previously (Lewis and Muday, 2009) with minor modifications. Briefly, 5-d-old seedlings were transferred to new 1/2 MS agar plates. The root–shoot junctions were covered with agar with a final concentration of 100nM [^3^H]IAA. Seedlings were then placed upside down and incubated in the dark for 10h. After incubation, a 5mm section of the root close to the agar block was discarded, and the remaining root tissue was harvested and balanced in liquid scintillation solution (Beckman Coulter) for 30min. The assays were then determined using a Beckman Coulter Scintillation System (Model LS6500).

### Quantification analysis of IAA by gas chromatography-mass spectrometry-selected ion monitoring (GC-MS-SIMS)

Quantification of IAA was conducted according to a method described previously (Gao *et al.*, 2013) with minor modifications. Briefly, 0.1g of root tips (fresh weight) of wild type and *tic-2* were immediately frozen in liquid nitrogen at dawn and dusk. Endogenous IAA was extracted, purified, methylated by a stream of diazomethane gas, resuspended in 100 μl of ethyl acetate, and then analysed by GC-MS-SIMS (GCMS-QP2010 Plus equipped with a HP-5MS column; Shimadzu).

## Results

### A *TIC* mutant reduces root meristem size in a circadian clock-independent manner

**Fig. 1.**
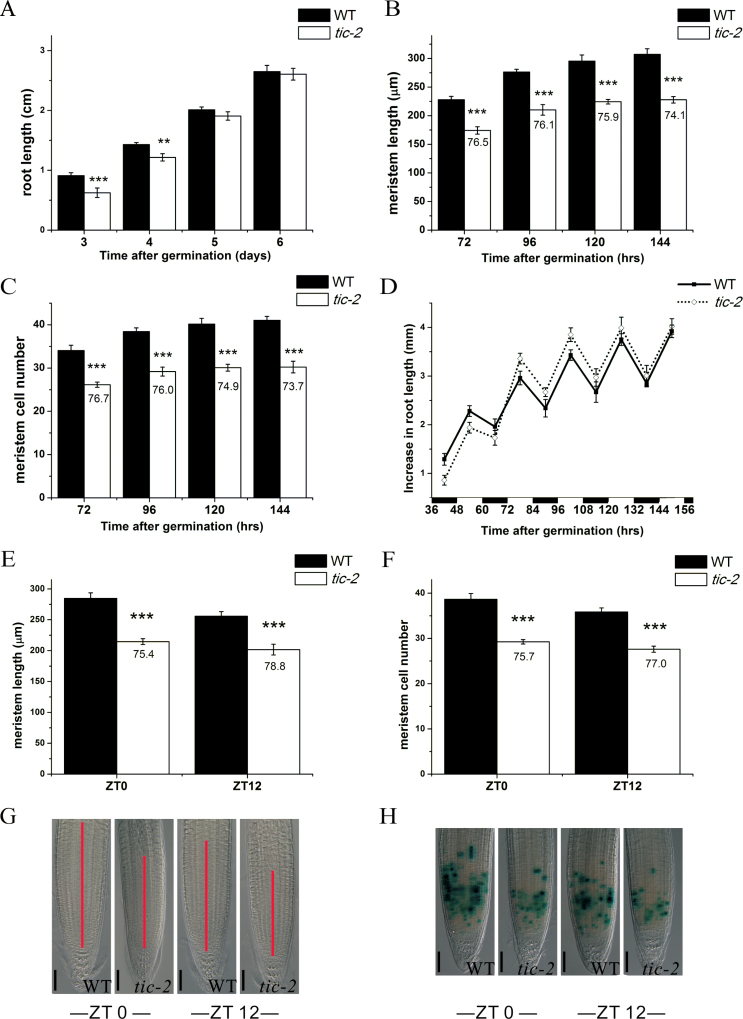
Reduced root meristem size in *tic-2* mutant plants. (A–C) Root lengths (A), root meristem lengths (B), and root meristem cell number (C) of 3–6-d-old wild-type (WT) and *tic-2* (ZT 24) plants. (D) Increase in root length of wild-type and *tic-2* plants during the day and night. (E–G) Root meristem lengths (E), root meristem cell number (F), and root meristem (G) of 5-d-old wild-type and *tic-2* plants at dawn and dusk. The red lines indicate the root meristem region. (H) GUS staining of *CycB1;1::GUS* at dawn and dusk in 5-d-old wild-type and *tic-2* plants. Bars, 50 µm (G, H). Data shown are means ±standard error of the mean (SEM) (*n*>30). Asterisks represents statistical significance (Student’s *t*-test, **P<0.01, ***P<0.001). The numbers in (B), (C), (E), and (F) are the percentage of root meristem size of *tic-2* compared with wild-type plants. (This figure is available in colour at *JXB* online.)

It has been reported that *TIC* is involved in JA-mediated root development. However, a 10-d-old *tic-2* mutant had a similar root length to that of wild type in the absence of exogenous methyl jasmonate (Shin *et al.*, 2012). To further investigate the function of *TIC* in root elongation, we measured the root length of *tic-2* and wild-type plants. Our data showed that primary root elongation was retarded in the *tic-2* mutant at either 3 or 4 d after germination (DAG), whereas no obvious inhibition was observed after 4 DAG in the wild type (Fig. 1A), indicating a role of *TIC* in early root elongation. As many reports show that a shorter root length is usually associated with changes in the meristem region (Fernandez-Marcos *et al.*, 2011; Zhang *et al.*, 2011), we further focused our observations on the root meristem zone. Significantly reduced meristem length and cell number were assayed in *tic-2* roots at 3, 4, 5, and 6 DAG compared with those of wild-type plants (Fig. 1B, C), suggesting that *TIC* affects root elongation by modulating meristem cell number. Interestingly, elongation zone length was not affected, and cell sizes in the maturation zone were larger in the *tic-2* mutant than in the wild-type seedlings during 3–6 DAG (Fig. S1 at *JXB* online).

As *TIC* is a circadian clock regulator, the photoperiod may also operate in this process. Thus, we grew the plants under several different photoperiods (8L/16, 12L/12D, 16L/8D, and 24L/0D) and measured both root meristem length and cell number. No significant difference for inhibited root meristem size and decreased cell number in *tic-2* plants was detected under any of the tested photoperiods (Fig. S2 at *JXB* online). Furthermore, we tested whether root elongation was affected in *tic-2* plants at dawn and dusk because root elongation rates have previously been reported to exhibit an oscillation (Fisahn *et al.*, 2012). For this purpose, primary root elongations both during the day and night were examined in wild-type and *tic-2* seedlings. We found that *tic-2* had a similar rhythmic root elongation to wild-type plants (Fig. 1D). When meristem length and cell number were examined in the roots of wild-type and *tic-2* plants at either dawn (ZT0) or dusk (ZT12), *tic-2* plants had a shorter meristem length and fewer meristem cells at both dawn and dusk compared with those of wild-type plants (Fig. 1E–G).

The decreased meristem size could be due to the loss of meristematic cell division potential. This can be analysed with *CycB1;1::GUS*, an excellent marker for cells undergoing mitosis to monitor cell-cycle progression (Colon-Carmona *et al.*, 1999). Thus, we crossed it with *tic-2* and assayed *CycB1;1* activity of the resultant line by GUS staining. The percentage of GUS-stained cells in the root meristem was significantly reduced in *tic-2* plants compared with that in wild-type plants at both dawn and dusk (Fig. 1H), suggesting that the mutation in *TIC* reduced the competence of meristematic cells to divide. An alternative explanation for the decreased meristem size could be a reduced stem cell niche activity. We obtained *tic-2 QC25::GUS* by crossing *tic-2* with the QC-expressed promoter trap *QC25::GUS*, and showed that about 16% of *tic-2 QC25::GUS* plants had extra QC cells, increased columella cell numbers, or additional stem cell tiers (Fig. S3 at *JXB* online), implying that *TIC* also functions in stem cell niche potential.

### *TIC* promoter is highly active in root meristem

**Fig. 2.**
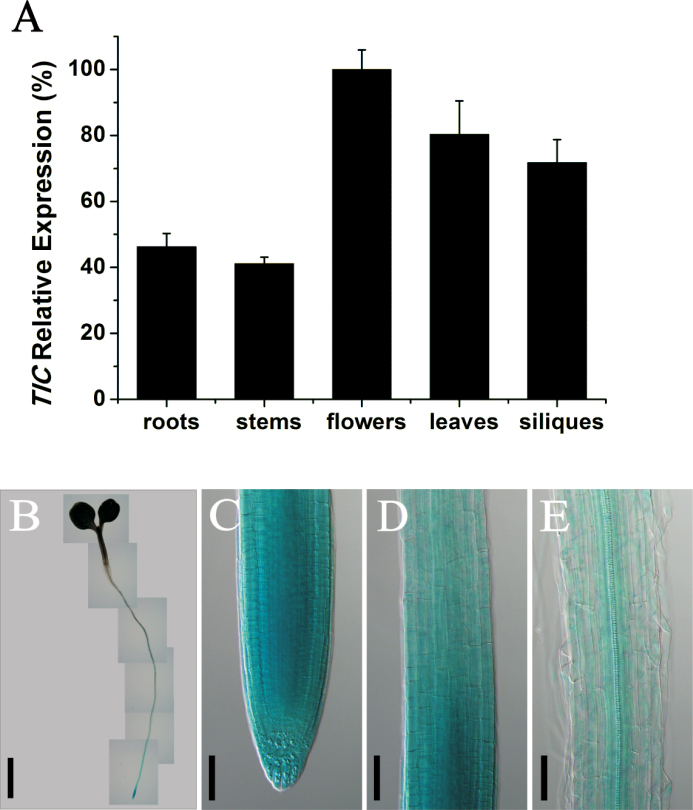
Expression pattern of *TIC*. (A) Quantitative RT-PCR analysis of *TIC* expression in 30-d-old wild-type plants. The transcript level in the flowers was set to 1. Data shown are means ±SEM. (B–E) Detection of GUS activity in 5-d-old *TIC::GUS* seedlings (ZT0) in the whole plant (B), meristem (C), elongation zone (D), and maturation zone (E). Bars, 2mm (B); 50 µm (C–E). (This figure is available in colour at *JXB* online.)

To understand the role of *TIC*, the expression pattern of this gene was initially analysed with quantitative RT-PCR in different organs including root, stem, flower, leave, and green silique. We found that *TIC* was universally expressed in all tested organs, with the highest level in flowers (Fig. 2A). To examine its expression profile in the root in detail, we obtained transgenic lines with a GUS reporter under the control of the *TIC* promoter. Our GUS staining data showed that the *TIC::GUS* lines had a gradient staining with the strongest activity in the root meristem (Fig. 2B–E), consistent with the function of *TIC* in root meristem.

### Decreased auxin accumulation is responsible for the reduced meristem size of *tic-2* roots

**Fig. 3.**
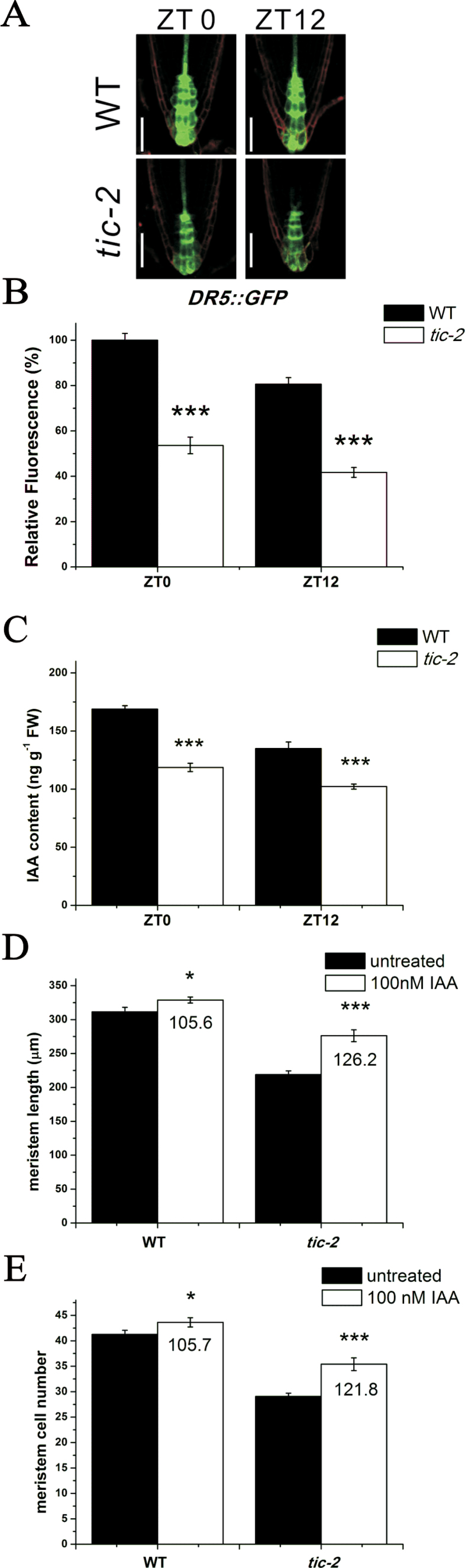
Decreased auxin accumulation in the roots of *tic-2* plants. (A) Expression of *DR5::GFP* at dawn and dusk in 5-d-old wild-type and *tic-2* plants. Bars, 50 µm. (B) Quantification of *DR5::GFP* fluorescence at dawn and dusk in 5-d-old wild-type and *tic-2* Plants. The fluorescence of wild-type plants at dawn was set to 100%. (C) IAA levels at dawn and dusk in roots of 5-d-old wild-type and *tic-2* plants. (D, E) Root meristem lengths (D) and root meristem cell number (E) of wild-type and *tic-2* plants in response to exogenous IAA at dawn. Data shown are means ±SEM (*n*>30). Asterisks represent statistical significance (Student’s *t*-test, *P<0.05, ***P<0.001). The percentage of root meristem size of IAA-treated seedlings compared with untreated seedlings is indicated below the corresponding bars, and these differences were statistically significant. (This figure is available in colour at *JXB* online.)

The fact that many mutants with low auxin levels have a reduced meristem size indicates that auxin is essential for the maintenance of root meristem (Sabatini *et al.*, 1999; Zhou *et al.*, 2010; Chen *et al.*, 2011*a*). Defective root meristem patterning in *tic-2* plants raised the question of whether auxin signalling was affected in *tic-2* mutants. Thus, we crossed *tic-2* plants with an auxin-responsive *DR5::GFP* marker line, whose pattern of expression provides reliable information on auxin accumulation and distribution (Ulmasov *et al.*, 1997). The fluorescence intensity of *DR5::GFP* in the roots of *tic-2* plants was dramatically decreased compared with that of wild-type roots at both dawn and dusk (Fig. 3A, B). Then, we directly measured endogenous IAA levels in the roots of wild-type and *tic-2* plants by GC-MS. Consistent with the observations for *DR5::GFP*, IAA levels were significantly lower in *tic-2* plants than in wild-type plants at both dawn and dusk (Fig. 3C), suggesting that the decreased auxin level could be responsible for the changed meristem size of *tic-2* roots. This was further reinforced by exogenous auxin application. We treated 4-d-old *tic-2* and wild-type seedlings with 100nM IAA for 24h and then measured root meristem size. Our data showed that root meristem size and cell number in *tic-2* plants were remarkably increased by IAA application, indicating that exogenous auxin can partially rescue the development defect present in the root meristem of *tic-2* mutants. Similar results was obtained at both dawn and dusk (Figs 3D, E and S4 at *JXB* online).

Furthermore, we investigated whether auxin regulated *TIC* expression. We found that *TIC* expression was unchanged in IAA-treated roots compared with untreated control (Fig. S5A at *JXB* online). This conclusion was further evidenced with *TIC::GUS* lines treated with or without IAA (Fig. S5B).

### Acropetal auxin transport is reduced in *tic-2* plants

**Fig. 4.**
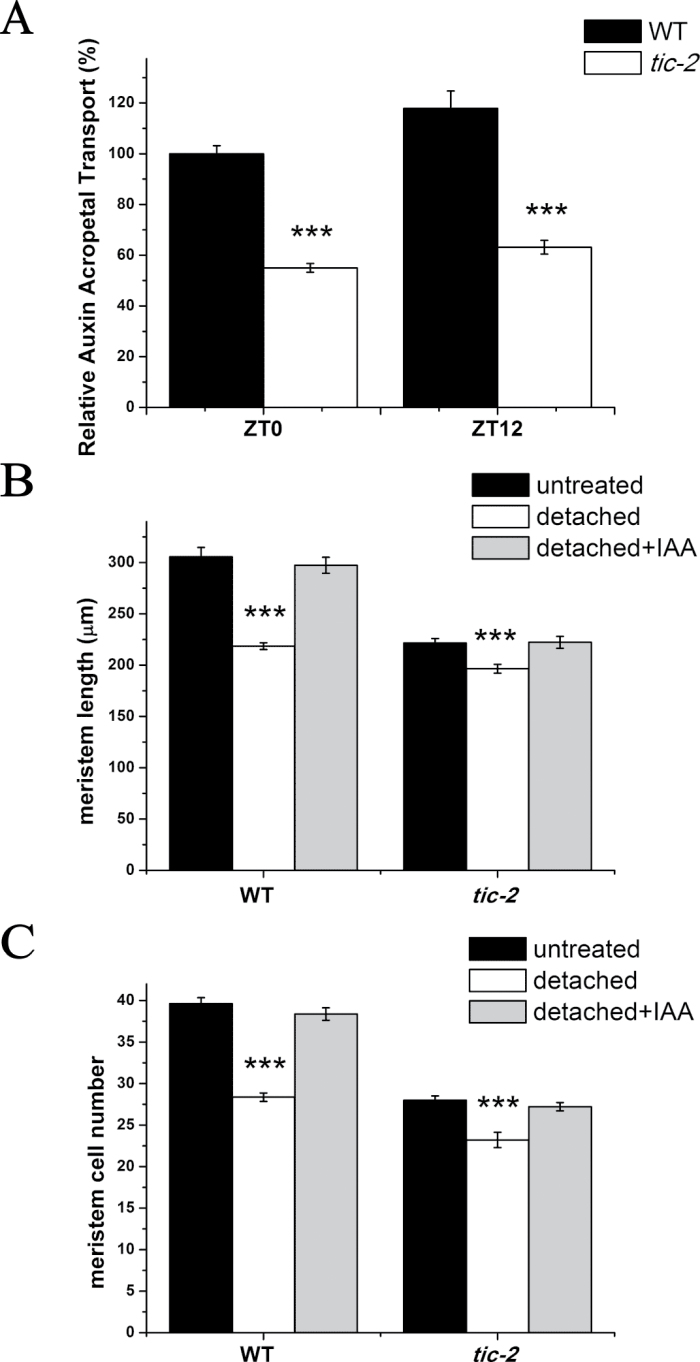
Reduced acropetal auxin transport in *tic-2* plants. (A) Relative acropetal auxin transport in 5-d-old wild-type and *tic-2* plants. The auxin transport of wild-type plants at dawn was set to 100%. Data shown are means ±SEM (two replicates, each with 10 seedlings). (B, C) Root meristem lengths (B) and root meristem cell number (C) of wild-type and *tic-2* plants in excision experiments at dawn. Data shown are means ±SEM (*n*>30) for (B) and (C). Asterisks represent statistical significance (Student’s *t*-test, ***P<0.001).

The differential auxin accumulation in the primary root requires an active auxin transport, and shoot auxin can be transported acropetally to the root by auxin transporters (Dai *et al.*, 2006). The above result that mutation in *TIC* decreased auxin accumulation in the roots implied that polar auxin transport might be affected in *tic-2* plants. Thus, we assayed auxin transport by applying [^3^H]IAA to the root–shoot junction and counting radiolabelled IAA in the root tip after 10h of incubation. A significant decrease in auxin movement in *tic-2* plants at both dawn and dusk was observed, suggesting that disruption of *TIC* caused a defect in acropetal auxin transport (Fig. 4A). These results suggested that the decreased auxin level in the root of *tic-2* plants could be, at least in part, due to reduced acropetal auxin transport.

We next examine whether removing plant aerial parts affected the root meristem, as it has been reported that auxin is synthesized mainly in the shoot apex and young leaves (Ljung *et al.*, 2001) and that shoot-derived auxin is necessary for root development (Friml *et al.*, 2003; Wisniewska *et al.*, 2006). The shoot parts of 4-d-old plants were excised and the remaining roots were incubated for 24h for examination of root meristem. Our results showed that shoot detachment in wild-type plants caused a significant decrease in both meristem length and cell number, resembling the phenotypes of *tic-2* plants. However, the *tic-2* mutant was less sensitive to shoot detachment (Fig. 4B, C). To further confirm that it was auxin from the aerial part that played an important role in maintaining root meristem size, we applied 1 μM IAA to the cut site and examined the phenotype. Consistent with our expectations, IAA application to the root–shoot junction restored the short-meristem phenotype (Fig. 4B, C). Similar results were obtained at both dawn and dusk (Figs 4B, C and S6 at *JXB* online).

In addition, we also assayed the expression of auxin synthesis genes by quantitative RT-PCR in the roots of both wild-type and *tic-2* plants. Among eight tested genes, the expression of *ASA1*, *ASB1*, *TAA1*, *TAR1*, *TAR2*, *YUC1*, and *YUC6* was downregulated in *tic-2* roots, suggesting that auxin biosynthesis was affected in *tic-2* plants (Fig. S7 at *JXB* online).

### *PIN* genes are downregulated in *tic-2*

**Fig. 5.**
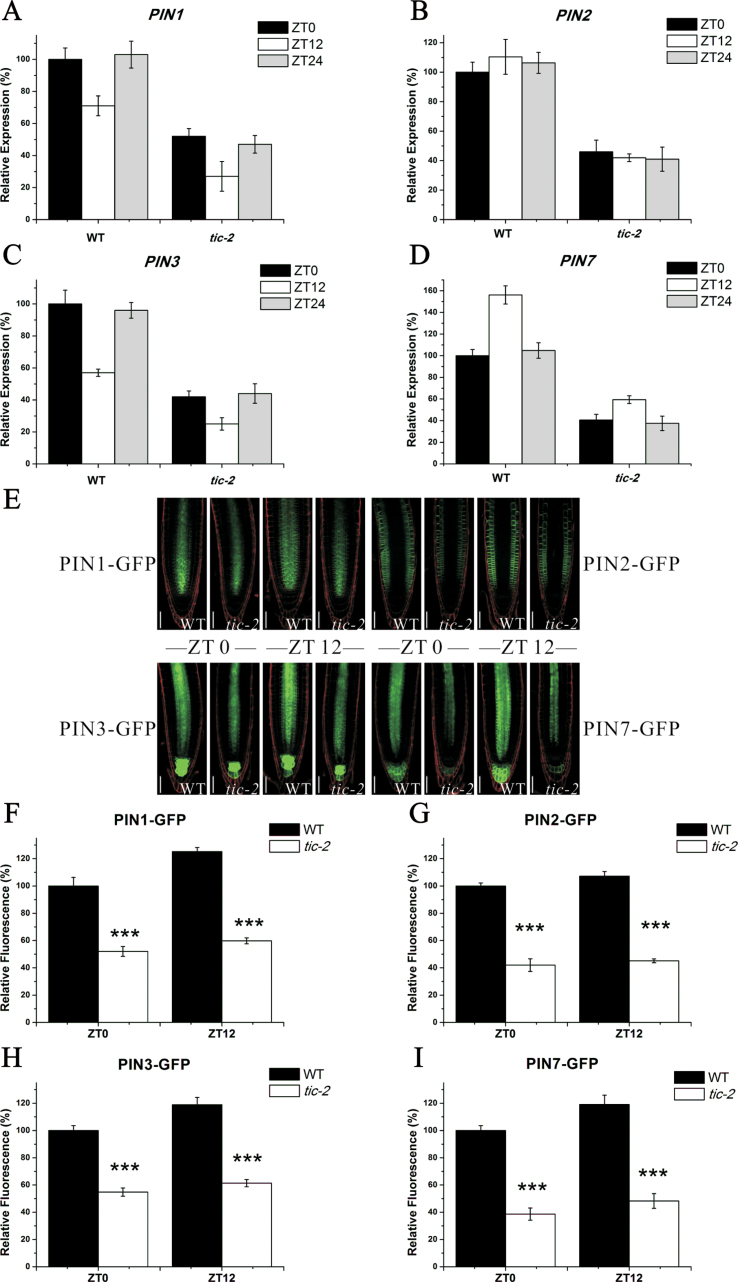
The mutation in *TIC* represses expression of *PIN*s. (A–D) Quantitative RT-PCR analysis of *PIN1* (A), *PIN2* (B), *PIN3* (C), and *PIN7* (D) in the roots of 5-d-old wild-type and *tic-2* plants. The transcript levels of wild-type plants at dawn were set to 100%. Data shown are means ±SEM. (E) Expression of *PIN1::PIN1-GFP*, *PIN2::PIN2-GFP*, *PIN3::PIN3-GFP*, and *PIN7::PIN7-GFP* in the roots of 5-d-old wild-type and *tic-2* plants at dawn and dusk. Bars, 50 µm. (F–I) Quantification of PIN–GFP fluorescence by image analysis of confocal sections. The fluorescence of wild-type plants at dawn was set to 100%. Data shown are means ±SEM (*n*>30). Asterisks represents statistical significance (Student’s *t*-test, ***P<0.001). (This figure is available in colour at *JXB* online.)

It has been documented that *PIN1*, *PIN2*, *PIN3*, nd *PIN7* play a critical role in controlling root meristem size (Blilou *et al.*, 2005; Dello Ioio *et al.*, 2008). Thus, auxin efflux *PIN*s may be also involved in the *TIC*-mediated changes in auxin level in the roots. To test this, we first analysed the expression levels of these genes by quantitative RT-PCR in the roots of both wild-type and *tic-2* plants and found that the transcript levels of these four *PIN*s were dramatically repressed in *tic-2* plants compared with those in wild-type plants (Fig. 5A–D). To further verify these in protein levels, four *PIN* reporter lines: *PIN1::PIN1-GFP*, *PIN2::PIN2-GFP*, *PIN3::PIN3-GFP* and *PIN7::PIN7-GFP* were crossed to *tic-2* plants and the resultant progenies homozygous for both *tic-2* and GFP reporters were selected for further confocal analysis. In agreement with the quantitative RT-PCR results, the fluorescence intensities for PIN1–GFP, PIN2–GFP, PIN3–GFP, and PIN7–GFP were remarkably reduced in *tic-2* plants (Fig. 5E). Our quantification of the respective fluorescence intensities revealed a reduction in GFP signals in the root tips of *tic-2* roots when compared with the same region in wild-type roots (Fig. 5F–I). Similar results were obtained at dawn and dusk (Fig. 5). Collectively, our data suggested that downregulation of *PIN* genes could be the cause of the reduced auxin transport in *tic-2* roots.

### Mutation of *TIC* represses expression of the *PLT* family

**Fig. 6.**
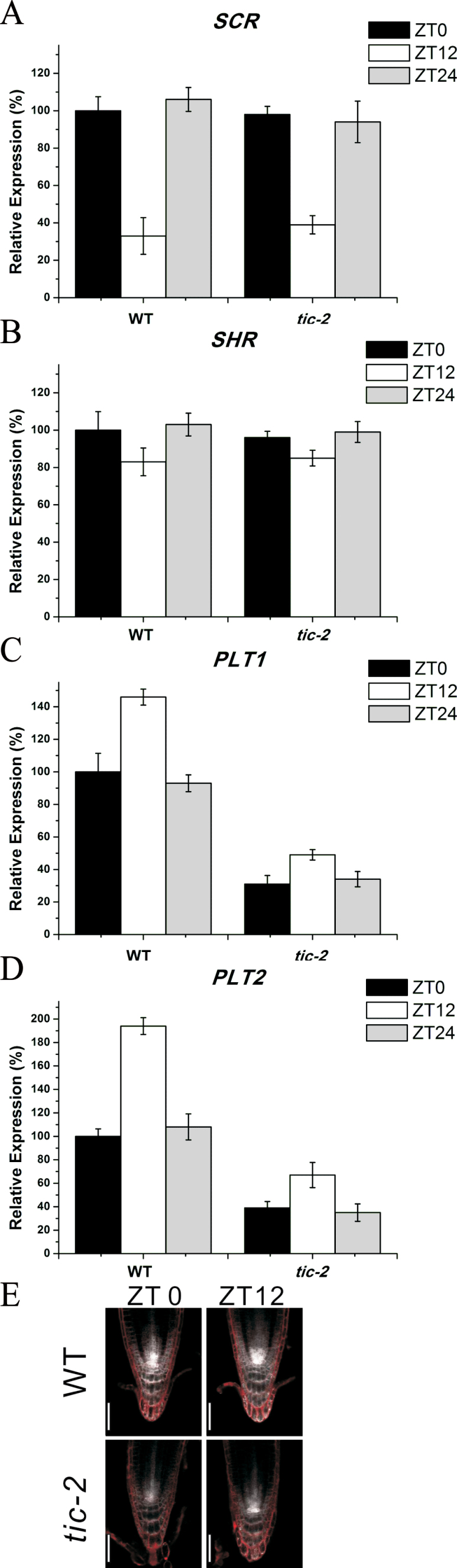
Expression of *SCR*, *SHR*, and *PLT*s in *tic-2* plants. (A–D) Quantitative RT-PCR analysis of *SCR* (A), *SHR* (B), *PLT1* (C), and *PLT2* (D) in the roots of 5-d-old wild-type and *tic-2* plants. The transcript levels of wild-type plants at dawn were set to 100%. Data shown are means ±SEM. (E) Expression of *PLT1::ERCFP* in the roots of 5-d-old wild-type and *tic-2* plants. Bars, 50 µm. (This figure is available in colour at *JXB* online.)

Maintenance of the root stem cell niche activity requires two parallel transcription factor pathways: the *SCR/SHR* pathway and the *PLT* pathway (Helariutta *et al.*, 2000; Lim *et al.*, 2000; Aida *et al.*, 2004; Galinha *et al.*, 2007). In our experiments, we first analysed the expression of the *SCR*/*SHR* genes using quantitative RT-PCR. No significant changes were assayed in *tic-2* roots compared with that in wild-type roots (Fig. 6A, B). In contrast to *SCR/SHR*, the expression of both *PLT1* and *PLT2* in *tic-2* plants was much lower than that in wild-type plants, demonstrating that *TIC* modulates the expression of *PLT*s (Fig. 6C, D). This was further verified using the *PLT1::ERCFP* marker line. The CFP fluorescence of *tic-2 PLT1::ERCFP* roots was dramatically reduced compared with that of the *PLT1::ERCFP* line (Fig. 6E). Combined with above data that a mutation in *TIC* results in low auxin level in the roots, these results suggested that *PLT1/2*, but not *SCR/SHR*, are downregulated by low auxin in the *tic-2* mutant for reduced meristem size at either dawn or dusk.

### *TIC* regulates meristem size independently of *MYC2*

**Fig. 7.**
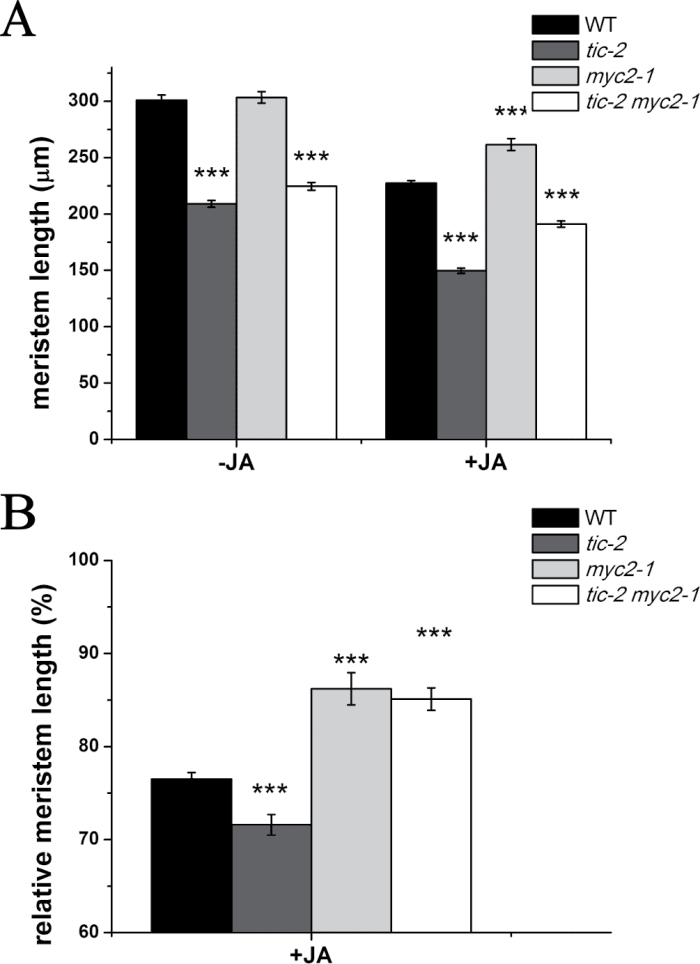
*TIC* mutation results in short root meristem size independent of *MYC2* at dawn. (A) Root meristem lengths of wild-type, *tic-2*, *myc2-1* and *tic-2 myc2-1* seedlings with or without exogenous application of JA at dawn. (B) Relative root meristem lengths of each genotype treated with JA compared with root meristem length without JA treatment at dawn. Data shown are means ±SEM (*n*>30). Asterisks represents statistical significance (Student’s *t*-test, ***P<0.001).

Recently, *TIC* was shown to be a negative regulator in JA signalling by repressing MYC2 protein accumulation (Shin *et al.*, 2012). It was also shown that JA inhibited root meristem activity in a *MYC2*-dependent manner (Chen *et al.*, 2011*b*). We predicted that the short root meristem zone in *tic-2* plants could be due to a high accumulation of MYC2. Thus, we crossed *tic-2* and *myc2-1* mutants (Boter *et al.*, 2004) and used the resultant *tic-2 myc2-1* plants for further analysis. However, in contrast to our expectations, both *tic-2* and *tic-2 myc2-1* mutants displayed a reduced meristem, whereas wild-type and *myc2-1* plants had a similar root meristem length when the plants were grown in the absence of exogenous JA (Fig. 7A), indicating that the shortened meristem size resulting from *TIC* mutation might be independent of *MYC2*. To see whether the repression effect of JA on meristem size could be altered in *tic-2* plants, 4-d-old seedlings of wild-type, *tic-2*, *myc2-1*, and *tic-2 myc2-1* plants were treated with 20 μM JA for 24h and the root meristem length was measured. Our experiments showed that *myc2-1* seedlings were less sensitive to JA for root meristem size compared with wild-type plants (Fig. 7), indicating the role of *MYC2* in JA-mediated root meristem size, as reported previously (Chen *et al.*, 2011*b*). In addition, *tic-2 myc2-1* seedlings were less sensitive, whereas *tic-2* seedlings were hypersensitive to JA (Fig. 7). This was because no MYC2 protein is expressed in *tic-2 myc2-1* plants and the high accumulation of MYC2 in *tic-2* is activated by JA. Similar results were obtained at both dawn and dusk, with a higher sensitivity of wild-type plants to exogenous JA in the morning than in the evening (Figs 7 and S8 at *JXB* online), consistent with a previous report (Shin *et al.*, 2012).

## Discussions

Previous reports on *TIC* have concentrated mainly on its function in the regulation of the circadian clock (Hall *et al.*, 2003; Ding *et al.*, 2007; Shin *et al.*, 2012). In this study, we demonstrated that *TIC* functions in the maintenance of post-embryonic root meristematic activity by regulating *PLT* expression, possibly via changes of auxin levels in the roots, uncovering a previously unknown role of *TIC* in the auxin–*PLT* loop to regulate root meristem development in *Arabidopsis*.

Plant growth and development rely on the activity of meristematic groups of undifferentiated cells that provide the tissues for new organ growth. The root meristem is established during embryogenesis, serving as the source for post-embryonic root development (Weigel and Jurgens, 2002). In this study, we have provided convincing evidence showing that loss of function of *TIC* leads to reduced meristem length and cell number with a decreased *CycB1;1::GUS* activity, suggesting that mutation in *TIC* reduced meristem size by affecting the meristematic cell division potential.

Root growth is determined by meristem cell division and subsequently cell elongation/differentiation (Dello Ioio *et al.*, 2007). Consistent with the shortened root meristem size, *tic-2* seedlings also had a shorter root length in the first 4 d after germination, whereas there was no difference in root length between *tic-2* and wild-type plants after 4 DAG. This was due to the longer longitudinal length of the root maturation zone cells, while the root elongation zone was not affected in *tic-2* plants (Fig. S1). The trend of shorter root due to reduced meristem size and that of longer root caused by the larger maturation zone cells in *tic-2* plants reached an equilibrium point, resulting in similar root lengths of both *tic-2* and wild-type plants after 4 DAG.

Auxin is essential for root meristem development, and shoot-derived auxin transport is one of the most important sources for auxin accumulation in the root apex (Blilou *et al.*, 2005; Ding and Friml, 2010). It has been reported that the root meristem size is determined by an auxin ‘reﬂux’ loop mediated mainly by auxin efflux carriers (Dello Ioio *et al.*, 2008). In addition, PIN-mediated auxin fluxes are sufficient to maintain a stable auxin maximum in the root based on computational modelling (Grieneisen *et al.*, 2007). In response to a *PIN*-mediated auxin maximum, *PLT* expression becomes restricted to define the stem cell region (Aida *et al.*, 2004), and in turn, *PLT*s regulate root-specific *PIN* expression to stabilize the auxin maximum and niche location (Blilou *et al.*, 2005; Galinha *et al.*, 2007; Pinon *et al.*, 2012), making the auxin–*PLT*–*PIN* feedback loop that controls root meristem maintenance. In our study, the results showed that the short-meristem defect in *tic-2* plants was mediated by reduced auxin accumulation due to repressed expression of *PIN*s, resulting in downregulation of *PLT1*/*2* for decreased stem cell niche activity.

Another phytohormone, JA, also participates in root growth regulation. The expression of JA-responsive genes is regulated by the basic helix–loop–helix transcription factors (*MYC*s), of which three homologues (*MYC2*, *MYC3*, and *MYC4*) have been well characterized (Fernandez-Calvo *et al.*, 2011; Niu *et al.*, 2011). Although these three members show redundant function in response to JA, they also display specific functions (Fernandez-Calvo *et al.*, 2011). Both *MYC3* and *MYC4* are expressed strongly in aerial parts but weakly in the roots of young seedlings, while MYC2 plays an important role in the root. MYC2 participates in JA-mediated inhibition of root growth and root meristem development by directly repressing *PLT* expression. However, a *myc2-2* mutant displayed a normal *PLT* expression and root meristem in the absence of JA, and the effect of JA on *PLT* expression is independent of the auxin pathway (Chen *et al.*, 2011*b*). Furthermore, TIC has been shown to represses MYC2 protein accumulation in JA signalling (Shin *et al.*, 2012). Our data showed that *TIC* regulates meristem size though the auxin–*PLT* loop in the absent of JA, and that the *myc2-1 tic-2* mutant had a reduced meristem comparable to that of *tic-2* mutants. Conversely, *myc2-1 tic-2* mutants acted similarly to *myc2-1* in the present of exogenous JA, indicating a complex regulation of root meristem by *TIC/MYC2* in different conditions (with/without JA). While *TIC* repressed the inhibition of JA on root growth in a *MYC2*-dependent manner, *MYC2* might not be necessary for the *TIC*-mediated root meristem development via the auxin–*PLT* loop.

## Supplementary data

Supplementary data is available at *JXB* online.

Supplementary Table S1. List of primers used in this study.

Supplementary Fig. S1. Mutant in *TIC* displays an unaffected elongation zone and longer maturation zone cells.

Supplementary Fig. S2. Reduced root meristem of *tic-2* in different photoperiods.

Supplementary Fig. S3. Root stem cell niche potential is affected in *tic-2* plants.

Supplementary Fig. S4. IAA application experiments at dusk.

Supplementary Fig. S5. Expression of *TIC* is not affected by exogenous auxin.

Supplementary Fig. S6. Aerial parts excision experiments at dusk.

Supplementary Fig. S7. Expression of auxin biosynthesis genes in *tic-2* plants.

Supplementary Fig. S8. *TIC* mutation results in short root meristem size independent of *MYC2* at dusk.

Supplementary Data

## Abbreviations:

CFP: cyan fluorescent protein
DAG: days after germination
GC-MS-SIMS: gas chromatography-mass spectrometry-selected ion monitorin
GFP: green fluorescent protein
GUS: β-glucuronidase
JA: jasmonic acid
MS: Murashige and Skoog
QC: quiescent centre
RT-PCR: real-time-PCR
SEM: standard error of the mean.
