# Supplementary material for: TIME FOR COFFEE controls root meristem size by changes in auxin accumulation in Arabidopsis
Source: J Exp Bot. 2013 Nov 25;65(1):275–86. doi: 10.1093/jxb/ert374 (PMC3883298; doi:10.1093/jxb/ert374)
Supplement: Supplementary Data [file supp_65_1_275__index.html]

 TIME FOR COFFEE controls root meristem size by changes in auxin accumulation in Arabidopsis — TIME FOR COFFEE controls root meristem size by changes in auxin accumulation in Arabidopsis — Supplementary Data 

# *TIME FOR COFFEE* controls root meristem size by changes in auxin accumulation in *Arabidopsis*

## Supplementary Data

Data files

**Files in this Data Supplement:**

- Supplementary Data - Supplementary Data
