# Supplementary material for: TIME FOR COFFEE controls root meristem size by changes in auxin accumulation in Arabidopsis
Source: J Exp Bot. 2013 Nov 25;65(1):275–86. doi: 10.1093/jxb/ert374 (PMC3883298; doi:10.1093/jxb/ert374)
Supplement: Supplementary Data [file supp_ert374_jexbot108944_file001.pdf]

Supplemental Data

TIC controls root meristem size by changes of auxin accumulation in *Arabidopsis*

Li-Wei Hong[1], Da-Wei Yan[1], Wen-Cheng Liu[1], Hong-Guo Chen[2] & Ying-Tang Lu[1]*

[1] College of Life Sciences, Wuhan University, Wuhan 430072, China

[2] College of Chemistry and Biology, Hubei University of Science and Technology, Xianning 437100, Hubei Province, China

Correspondence:

Ying-Tang Lu; Fax: 86-27-68753551; E-mail: yingtlu@whu.edu.cn

Email addresses of other authors:

Li-Wei Hong: liweihong@whu.edu.cn

Da-Wei Yan: pectin@gmail.com

Wen-Cheng Liu: liuwencheng@whu.edu.cn

Hong-Guo Chen: chhg1969@163.com

Supplementary table S1 List of primers used in this study.

TIC-proF	TCCCCCGGGGGGCCTAATCCATGTCACTATCATTC
TIC-proR	TCCCCCGGGCTTCTTCTTCTTCTTCTCTAATGCTTC
WOX5-rtF	GATTGTCAAGAGGAAGAGAAGGTGA
WOX5-rtR	AGCTTAATCGAAGATCTAATGGCG
SHR-rtF	CGCTCAACGAGCTCTCTTCT
SHR-rtR	ATGGTTCGGTAGCATCGTTC
SCR-rtF	ATTAGCGGTTGGAGG
SCR-rtR	ACTAAGAACGAGGCGT
PIN1-rtF	GGTGGTGGTCGGAACTCTAAC
PIN1-rtR	TAATGAAACCTCCCAGCTCCAG
PIN2-rtF	TTACTCCGTTCAATCGTCAC
PIN2-rtR	ACGCCTTTAGAAGACTGAAG
PIN3-rtF	TCTTTGATTAGGTTCGGGTAACTC
PIN3-rtR	GCTCATGTGAAACTGGAACAAG
PIN7-rtF	TCAAACGCTTCTCGGAGATC
PIN7-rtR	CGGGAAACCCCATCATAGAG
PDF2-rtF	GTGTTTATGTCGCGGTGAAG
PDF2-rtR	GTTCTCCACAACCGCTTGGT
UBQ1-rtF	GCAGATCTTCGTGAAAACCTTGACC
UBQ1-rtR	GCACTTGCGGCAAATCATCTTATCC
TIC-rtF	GGAGCAGCTAATAGCATCTTGC
TIC-rtR	CAGATGCGGCAGCTATTGTA
PLT1-rtF	CCAAAGTGGTAGTGATTTATTGATT
PLT1-rtR	AGAATTCATTTTCTTCTTTTTGAGTC
PLT2-rtF	GCAGCCATACTTGGAGAAA
PLT2-rtR	TTCTTGGAATCAAAGCTTAAACCA
tic-2-LP	CTTAGGAGCTGAAGCGAAAATGG

tic-2-RP	GAATATGACGACGGAGGTGTAG
LB3	TAGCATCTGAATTTCATAACCAATCTCGATACAC
myc2-1-LP	ATGACTGATTACCGGCTACAACCA
myc2-1-RP	AACCGATTTTTGAAATCAAACTTGC
LBa1	TGGTTCACGTAGTGGGCCATCG
YUC1-rtF	ACCCAAAATACCCTTCCAAAA
YUC1-rtR	AGTGGGAAGCGTAGGACTCA
YUC4-rtF	TGGAGGTCAGCTTGGATCTT
YUC4-rtR	TTCCAAATGTTGATAGACCAAAAA
YUC6-rtF	CTCGTTGTCAGAGACGCTGT
YUC6-rtR	AACCAAAAGGAAACGGTCAA
TAA1-rtF	AGCAGAGCTGGAGAGCGTTGTG
TAA1-rtR	CTTCATGTTGGCGAGTCTCTCGAG
TAR1-rtF	TCAGGAAGGCTCCTCAGACATTGC
TAR1-rtR	ACGCTGGTCAGAGTTATGAGACACC
TAR2-rtF	AAGGTTGTGTCAGACAGTTGTGGG
TAR2-rtR	GGTTGTGGCTCAAAGACCCTGC
ASA1-rtF	GTAGAGAAGCTTATGAACATCGA
ASA1-rtR	GGTGCACCACTAACTGTTCCCAC
ASB1-rtF	GGGGAAGAGTCGTAGAGATGTCT
ASB1-rtR	CTGGCAGAGATTGTATGTGAAGC

Supplementary figure S1

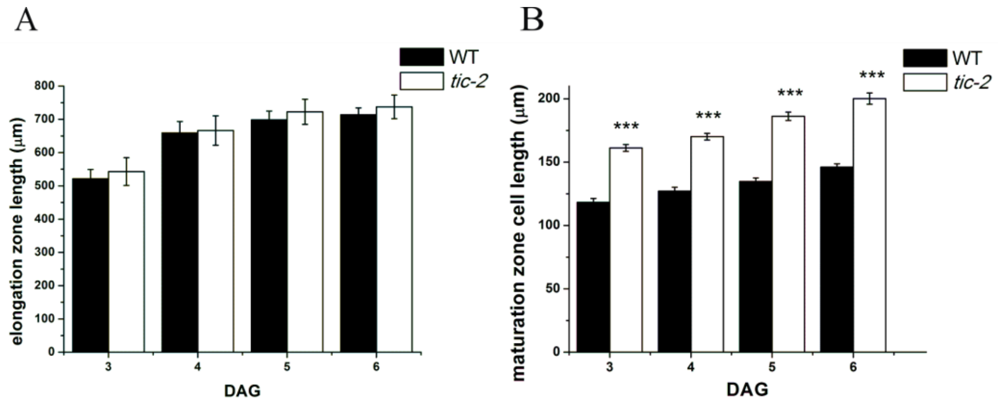

Fig. S1. The mutation in *TIC* displays an unaffected elongation zone and longer maturation zone cells. (A) Elongation zone lengths of 3 to 6-day-old wild-type and *tic-2*. (B) Cell lengths in the maturation zone of 3 to 6-days-old wild-type and *tic-2*. Data are shown as average ± SEM (n > 30). The asterisk represents statistical significance (Student's *t*-test, P < 0.001).

Supplementary figure S2

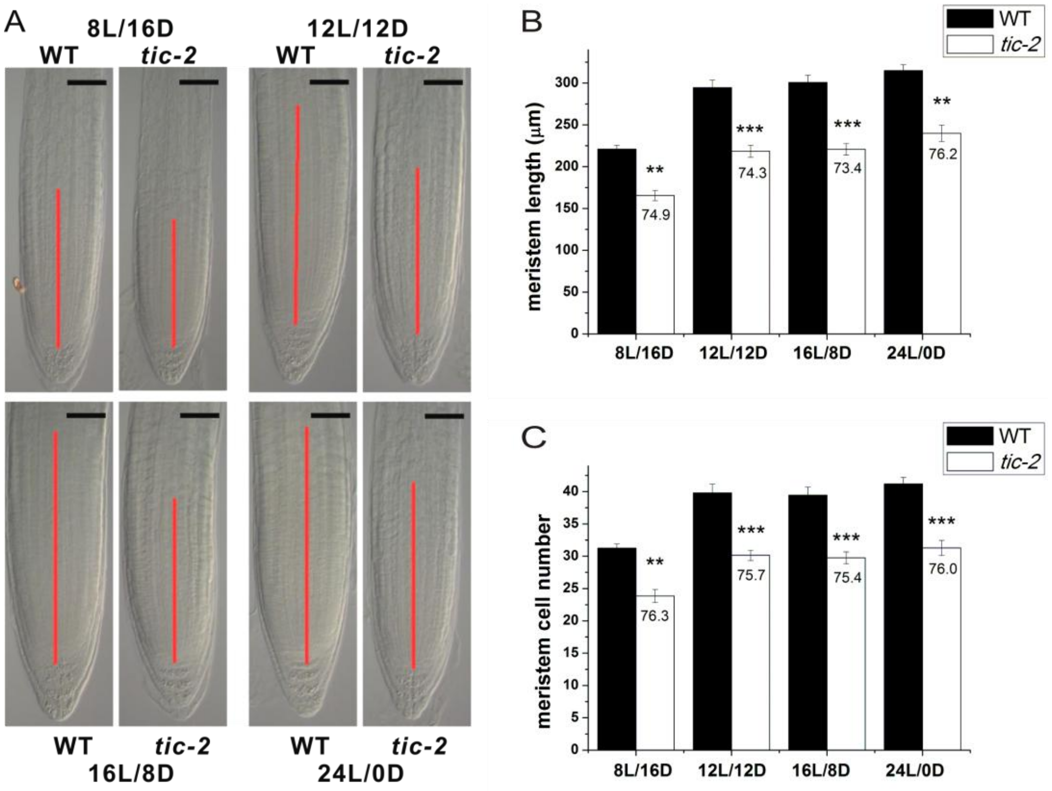

Fig. S2. Reduced root meristem of *tic-2* in different photoperiods. (A) Root meristem of 5-day-old wild-type and *tic-2* under different photoperiods (ZT 0). The lines indicate the root meristem region. Bars=50 μm. (B) Root meristem lengths of 5-day-old wild-type and *tic-2* under different photoperiods (ZT 0). (C) Root meristem cell number of 5-day-old wild-type and *tic-2* under different photoperiods (ZT 0). Data shown are average ± SEM (n > 30). The asterisk represents statistical significance (Student's *t*-test, P<0.01 or 0.001 respectively). The numbers in B and C are the percentage of root meristem size of *tic-2* compared with wild-type.

Supplementary figure S3

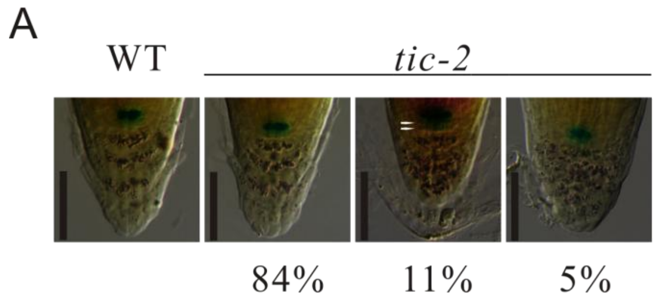

Fig. S3. Root stem cell niche potential is affected in *tic-2*. (A) GUS staining of *QC25::GUS* (blue) in the roots of 5-day-old wild-type and *tic-2* seedling performed with Lugol staining (dark brown). White arrow indicates columella stem cell (CSC). Bars=50 μm.

Supplementary figure S4

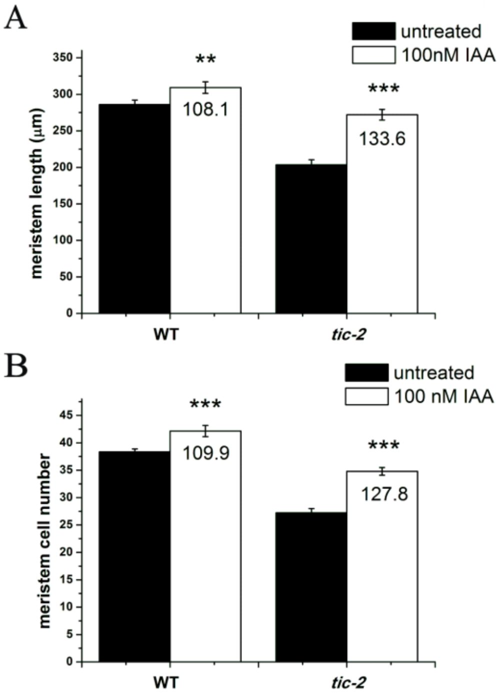

Fig. S4. IAA application experiments at dusk. (A) Root meristem lengths of wild-type and *tic-2* in response to exogenous IAA at dusk. (B) Root meristem cell number of wild-type and *tic-2* in response to exogenous IAA at dusk. Data shown are average ± SEM (n > 30). The asterisk represents statistical significance (Student's *t*-test, P<0.01 or 0.001 respectively). The percentage of root meristem size of IAA-treated seedlings compared with untreated seedlings is indicated below the corresponding bars, and the difference has statistical significance.

Supplementary figure S5

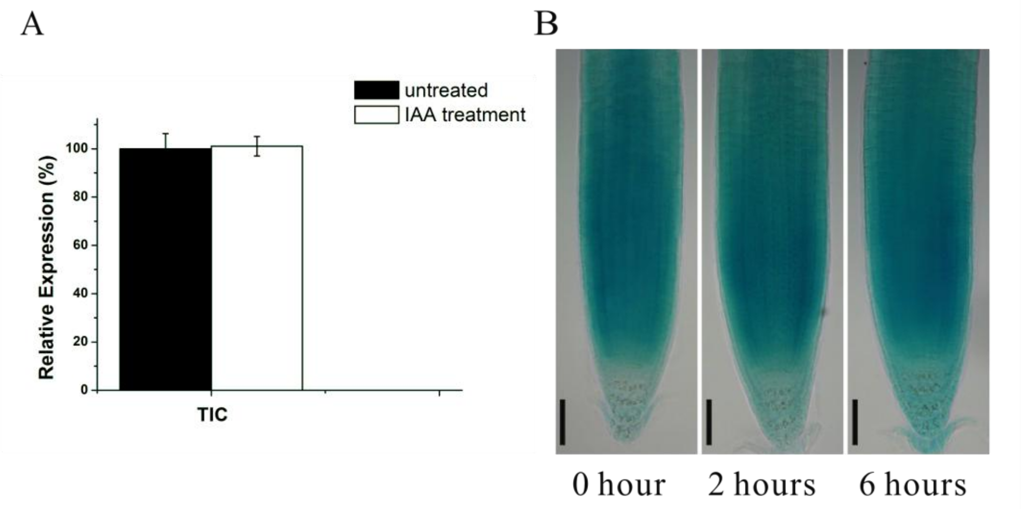

Fig. S5. Expression of *TIC* is not affected by exogenous auxin. (A) Quantitative RT-PCR analysis of *TIC* in 5-day-old wild-type roots treated by 5 μM IAA for 6 hours (ZT 0). The transcript level of untreated wild-type was set to 1. Data are shown as average ± SEM. (B) GUS staining of *TIC::GUS* in the roots of 5-day-old seedling treated by 5 μM IAA (ZT 0). Bars=50 μm.

Supplementary figure S6

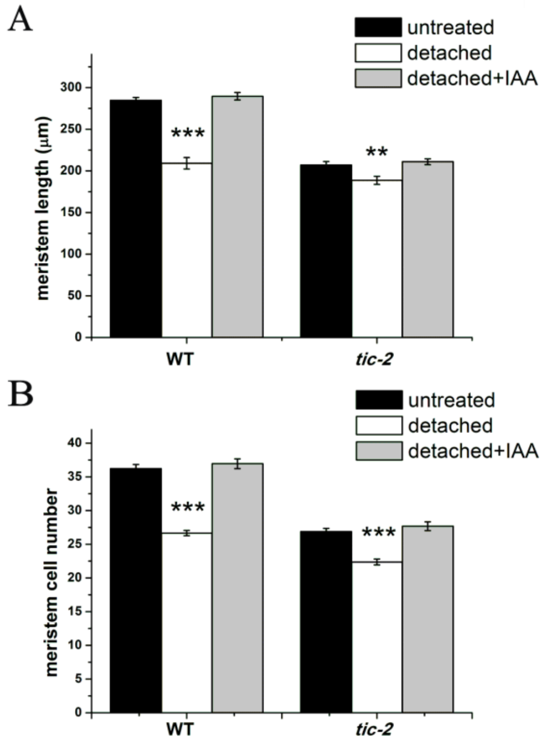

Fig. S6. Aerial parts excision experiments at dusk. (A) Root meristem lengths of wild-type and *tic-2* in excision experiments at dusk. (B) Root meristem cell number of wild-type and *tic-2* in excision experiments at dusk. Data shown are average ± SEM (n > 30) for (B) and (C). The asterisk represents statistical significance (Student's *t*-test, P <0.01 and 0.001, respectively).

Supplementary figure S7

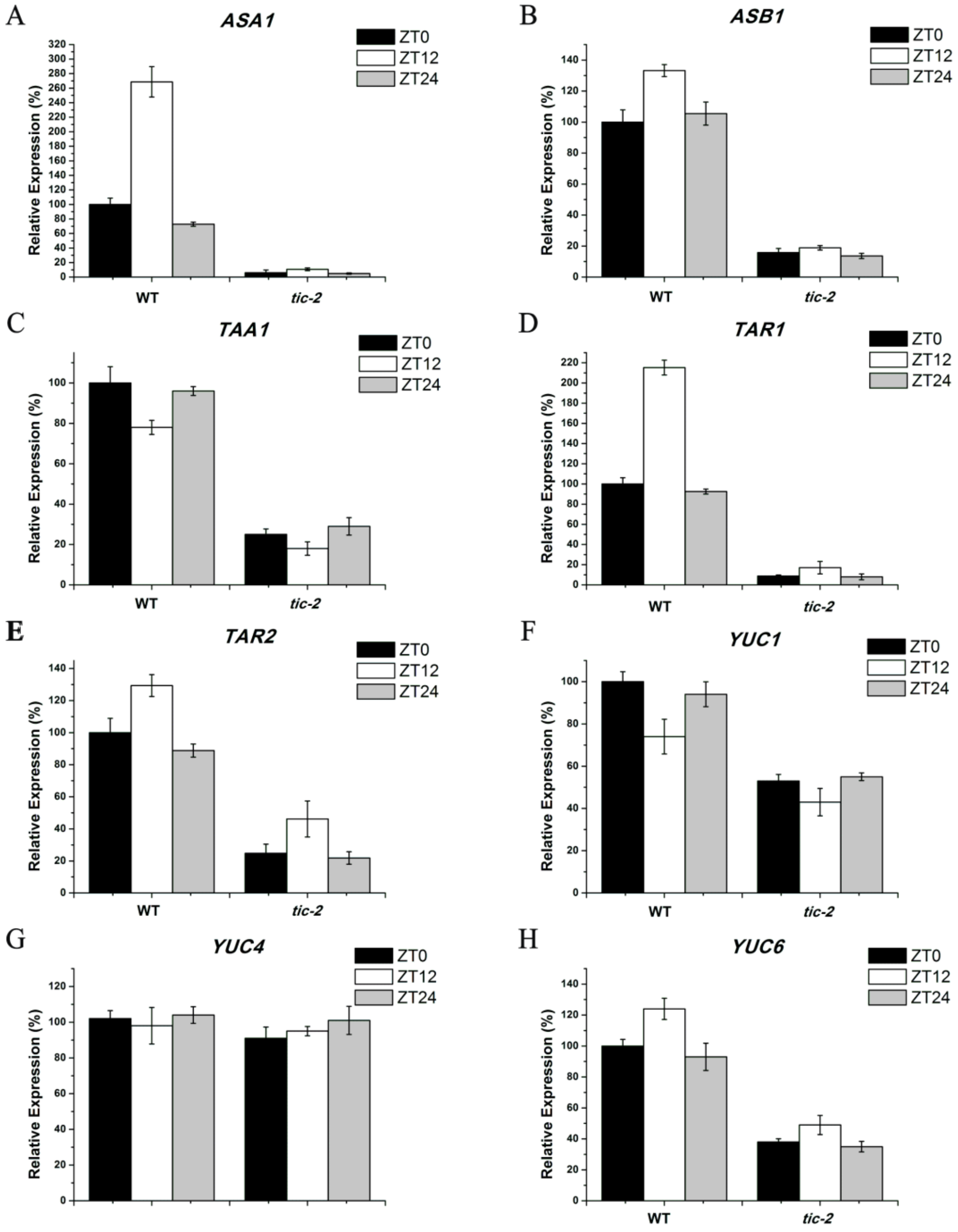

Fig. S7. Expression of auxin biosynthesis genes in *tic-2*. (A)-(H) Quantitative RT-PCR analysis of *ASA1* (A), *ASB1* (B), *TAA1* (C), *TAR1* (D), *TAR2* (E), *YUC1* (F), *YUC4* (G) and *YUC6* (H) in the roots of 5-day-old wild-type and *tic-2*. The transcript levels of wild-type at dawn were set to 1. Data shown are average ± SEM.

Supplementary figure S8

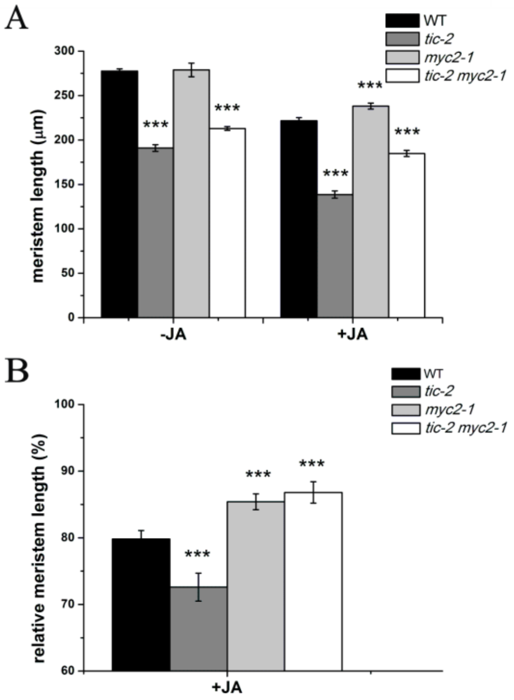

Fig. S8. *TIC* mutation results in short root meristem size independent of *MYC2* at dusk. (A) Root meristem lengths of wild-type, *tic-2*, *myc2-1* and *tic-2 myc2-1* seedlings with or without exogenous application of JA at dusk. (B) Relative root meristem lengths of each genotype treated with JA to root meristem length without JA treatment at dusk. Data shown are average ± SEM (n > 30). The asterisk represents statistical significance (Student's *t*-test, P < 0.001).
